# The aging venous system: from varicosities to vascular cognitive impairment

**DOI:** 10.1007/s11357-021-00475-2

**Published:** 2021-11-11

**Authors:** Andrea Ágnes Molnár, György László Nádasy, Gabriella Dörnyei, Bernadett Bettina Patai, Jordan Delfavero, Gábor Áron Fülöp, Angelia C. Kirkpatrick, Zoltán Ungvári, Béla Merkely

**Affiliations:** 1 Heart and Vascular Center, Semmelweis University, Városmajor Street 68, 1121 Budapest, Hungary; 2 Department of Physiology, Semmelweis University, Budapest, Hungary; 3 Department of Morphology and Physiology, Health Sciences Faculty, Semmelweis University, Budapest, Hungary; 4 Department of Traumatology, Military Hospital, Budapest, Hungary; 5 Vascular Cognitive Impairment and Neurodegeneration Program, Center for Geroscience and Healthy Brain Aging/Reynolds Oklahoma Center On Aging, Department of Biochemistry and Molecular Biology, University of Oklahoma Health Sciences Center, Oklahoma City, OK USA; 6 Department of Medicine, University of Oklahoma Health Sciences Center, Oklahoma City, OK USA; 7 Veterans Affairs Medical Center, 921 NE 13th Street, Oklahoma City, OK 73104 USA; 8 International Training Program in Geroscience, Doctoral School of Basic and Translational Medicine/Department of Public Health, Semmelweis University, Budapest, Hungary

**Keywords:** Veins, Aging, Venous insufficiency, Deep vein thrombosis, Varicose veins, GeroScience, Vascular cognitive impairment, Ageing

## Abstract

Aging-induced pathological alterations of the circulatory system play a critical role in morbidity and mortality of older adults. While the importance of cellular and molecular mechanisms of arterial aging for increased cardiovascular risk in older adults is increasingly appreciated, aging processes of veins are much less studied and understood than those of arteries. In this review, age-related cellular and morphological alterations in the venous system are presented. Similarities and dissimilarities between arterial and venous aging are highlighted, and shared molecular mechanisms of arterial and venous aging are considered. The pathogenesis of venous diseases affecting older adults, including varicose veins, chronic venous insufficiency, and deep vein thrombosis, is discussed, and the potential contribution of venous pathologies to the onset of vascular cognitive impairment and neurodegenerative diseases is emphasized. It is our hope that a greater appreciation of the cellular and molecular processes of vascular aging will stimulate further investigation into strategies aimed at preventing or retarding age-related venous pathologies.

## Introduction

Diseases that affect the circulatory system, including cardiovascular and cerebrovascular diseases, are the most common cause of death among older people in the developed countries [1]. The extent of human suffering, death, and economic damage caused by venous diseases in older adults is not far from that caused by arterial diseases [2–8]. The prevalence of several diseases of the arterial system exponentially increases with advancing age [9, 10]. Although several important venous diseases frequently appear at younger ages, their accumulation and progression with advanced age are also typical. Age is now accepted as an important independent risk factor of venous diseases [3–8, 11, 12]. Yet, the mechanisms and consequences of aging in veins are less extensively studied.

**Fig. 1:**
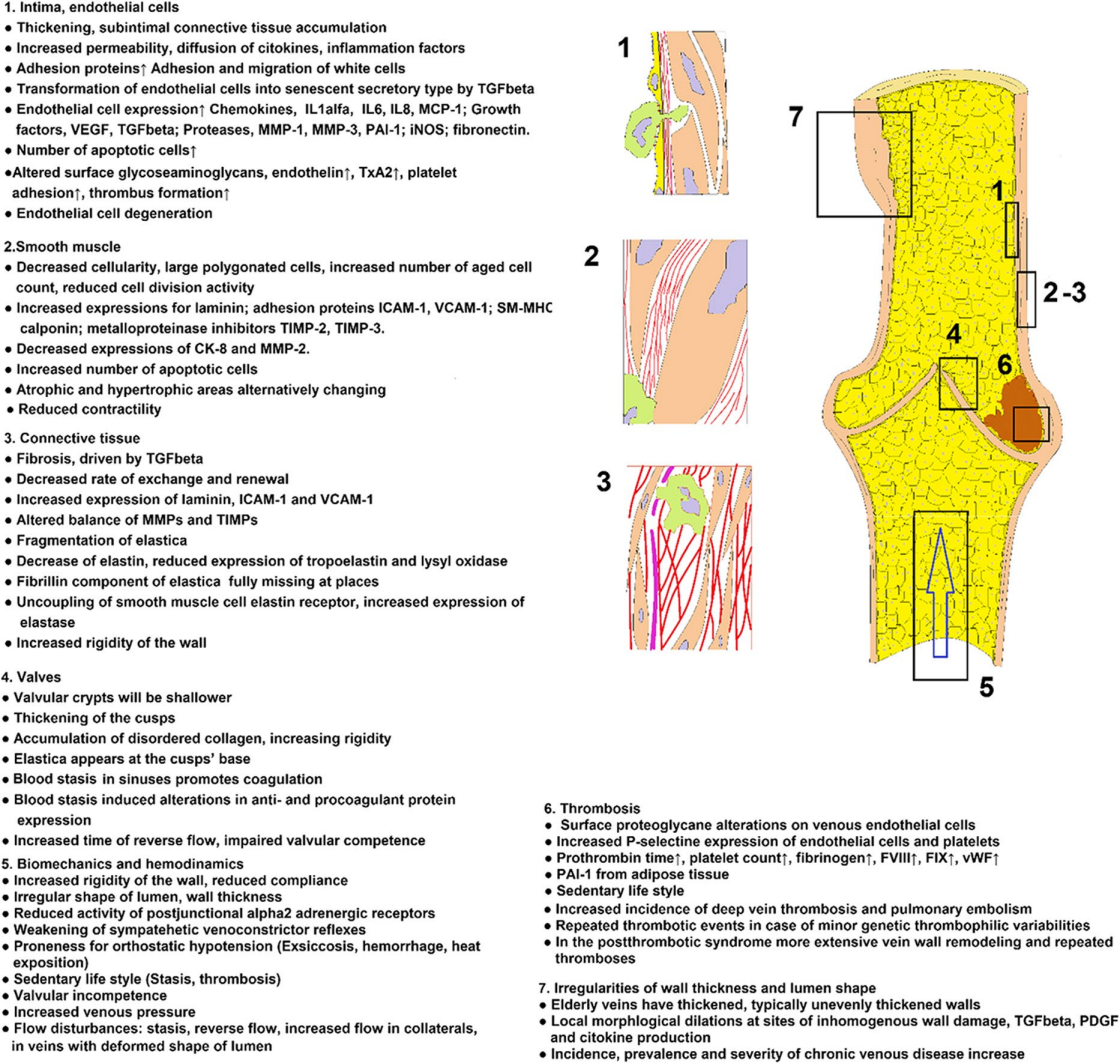
Aging processes identified in veins and their connections to venous pathology. For detailed description and references, see the corresponding chapters. Abbreviations: CK-8, Cytokeratin-8; FVIII, FIX, clot factors VIII and IX; ICAM-1, Intercellular adhesion molecule-1; iNOS, inducible nitrogen monoxide synthase; IL1α, IL6, IL8, interleukins 1alfa, 6, and 8; MCP-1, Monocyte chemotactic protein; MMP-1, MMP-2, MMP-3, Matrix metalloproteinases 1, 2, and 3; PAI-1; PDGF, platelet-derived growth factor; SM-MHC, Smooth muscle heavy chain; TIMP-2, TIMP-3, tissue inhibitor of matrix metalloproteinase 2 and 3; TxA2, thromboxane A2; VCAM-1, Vascular adhesion molecule-1; vWF, von Willebrand factor; TGFbeta, Transforming growth factor beta; VEGF, vascular endothelial growth factor

Aging of the arterial system and its pathological consequences have recently been reviewed in detail [9, 10, 13]. Cellular components of the venous wall are identical or close to those of the arteries and arterioles. It is assumed that many of the cellular and molecular aging processes that contribute to arterial aging impact also venous aging; however, there are important dissimilarities between aging of the venous and arterial systems and their pathological manifestations. This review discusses the shared processes of vascular aging and their putative contribution to age-related venous pathologies (Fig. 1) and highlights important differences between arterial and venous aging.

## Aging of veins and arteries: similarities and dissimilarities

There are important differences between the functional anatomy of veins and arteries which contribute to their differential sensitivity to age-related deterioration. Aging promotes atherosclerosis in the arteries [14, 15]. Although veins are exposed to the same circulating factors, they are free from atherosclerotic plaque development. This is primarily due to the markedly different hemodynamic environment in the arterial and venous circulations. Active and passive force-bearing elements of the venous wall are not stretched by the unceasing high wall stress caused by the high diastolic value of arterial pressure and its aggressive pulsatile alterations. Although similar age-related phenotypic alterations occur in the endothelial and smooth muscle cells (including a heightened state of inflammation), the hemodynamic environment and the altered response to injury of the vascular cells determine specific manifestations of vascular pathologies in the aged arteries and veins. Further, the venous endothelium is not subjected to the highest oxygen tension in the body like its arterial counterpart. Also, venous wall shear stress is much less than in the arteries due to the slower blood flow velocity. Yet, high wall stress can develop in the venous system. For example, the lower extremity of humans, to which the genetic adaptation still does not seem to be fully adequate, is a predilection site for disease [16]. The slower blood flow increases the danger of thrombotic processes, by elevating the probability of platelet and white blood cell attachment and also erythrocyte retention [17]. The blood reservoir function of veins requires extensive smooth muscle contraction to ensure appropriate vessel volume. The vein valves, which are tiny, sensitive anatomical structures, are frequently subjected to pathological processes [18, 19].

Endothelial cells of arteries are in direct contact with oxygenized blood, while high pressures of arterial blood extending into the inner layer of the artery wall exclude establishment of any microcirculation here. There is a much different situation in the venous wall. Exhausted blood is in contact with the inner layers but vasa vasorum microcirculation is possible in the wall.

The time course of venous pathologies is different from that of arterial diseases: many serious, advanced cases of venous disease accumulate at relative early age. Notwithstanding, there is a significant increase of venous pathologies with age [3, 4, 6, 7, 12, 20] induced by both the accumulation of age-dependent pathologic processes and by the increased sensitivity to inflammatory and thrombotic processes of the aged venous wall [21].

## Age-related cellular and morphological alterations in the venous system

### Endothelial aging

Among the cell types present in the vascular wall, endothelial cells are especially sensitive to the deleterious effects of aging [22–32]. There is increasing evidence that aging-induced phenotypic and functional alterations of endothelial cells contribute to the genesis of age-related venous pathologies, similar to the arterial system (Fig. 1). Endothelial dilation is depressed in aged veins [33], similar to aged arteries and arterioles [9, 23, 30, 34, 35]. Histologically endothelial cells in varicose saphenous vein specimens derived from older adults show progressive degeneration, and ultimately will be lost, exposing the basement membrane for platelets and clot factors and the medial layers for inflammatory protein permeation and migration of white cells [36]. The increased permeability of the endothelial layer in aged vessels promotes the entry of circulating inflammatory mediators (e.g., cytokines, factors that promote sterile inflammation) into the deeper layers of the vascular wall. With aging, vascular endothelial cells acquire a pro-inflammatory phenotype, which likely contributes to the development of venous diseases [37]. Varicose veins are characterized by up-regulation of iNOS [38], which is likely to promote the formation of peroxynitrite. Peroxynitrite is known to play multifaceted roles in vascular pathologies associated with aging, including activation of PARP-1 and promotion of mitochondrial dysfunction [39]. Also, an increasing ratio of aged endothelial cells undergo cellular senescence and exhibit a highly inflammatory senescent associated secretory phenotype (SASP). This phenotype is characterized by elevated expression and secretion of soluble signaling factors such as chemokines: IL8, MCP-1; interleukins, IL1α, IL6; growth factors: VEGF, TGFβ; proteases: MMP-1, MMP-3, MMP-10, PAI-1; further endothelial NO-synthase; and matrix components (fibronectin) [21]. Age-related alterations of adhesion proteins and glycosaminoglycans on the surface of endothelial cells promote platelet adhesion and thrombus formation, as well as adhesion and transmigration of leukocytes [21]. Increased presence of white blood cells in the aged vascular wall also contribute to an inflammatory microenvironment in aged veins [21]. Aged endothelial cells exhibit impaired resilience to oxidative stressors and are more sensitive to apoptosis induction. Accordingly, the number of apoptotic endothelial cells increases in veins of older individuals [40]. In the human saphenous vein, there is an intimal thickening with advanced age [41]. Molecular alterations of endothelial cells will be discussed in detail below.

### Smooth muscle aging

The aged venous media is scant of cellular components, smooth muscle cells are large, and cells obtained from aged donors are morphologically different from younger specimens when cultured [42]. Aged smooth muscle cells have a polygonate shape, and are frequently multinuclear. Their division activity ceases early, after about 10 passages [42]. In venous smooth muscle cell, sensitivity to growth factors substantially decreases with age [42]. The expression of laminin and of the adhesion proteins ICAM-1 and VCAM-1 increases with age [43]. In otherwise healthy saphenous vein graft samples, a positive correlation between age and the expressions of SM-MHC (Smooth Muscle Myosin Heavy Chain), calponin, TIMP-2 and TIMP-3 (Tissue Inhibitor of Metallo-Proteinase 2 and 3), and a negative correlation with CK-8 (Cytokeratin-8) and MMP-2 (matrix metalloproteinase-2) were found [44] demonstrating that a massive rearrangement of protein expression accompanies the aging process. There is a biologically significant phenotypic overlap with vascular smooth muscle cells isolated from pathologic varicose vein samples, in which expression of Bcl-2 (an apoptosis controlling protein, located at the outer mitochondrial membrane), MMP-1, MMP-2, TIMP-1, and TIMP-2 is dysregulated and proliferation, adhesion, and migration capacities are altered [45]. The number of apoptotic smooth muscle cells increases in the aged venous system as well as in the prevaricose-varicose venous wall [46–50]. In the affected venous networks, hypertrophic and atrophic areas are alternating giving a foundation for massive morphological deformations observed later in life [40, 47, 51].

### Connective tissue aging

Connective tissue alterations in the aged, sclerotic, and varicose vessels were historically the first to be recognized. Decreased cellularity, elastic tissue damage, and collagen accumulation are important components of both aging and varicose transformation of the venous wall [19, 52, 53]. Similar to other tissues, TGFβ appears to govern the pro-fibrotic phenotypic changes both in the aged veins and in the varicose vein wall [11, 54]. Turnover of connective tissue in the venous wall decreases with advancing age and with it the structure of elastic membranes and collagen bundles is altered. There is a higher level of MMP activity in aged veins, which contribute to the remodeling of the extracellular matrix. MMP activity is controlled by TIMP-s. In normal vascular tissue, there is a homeostatic balance between MMPs and TIMPs which is disturbed in pathological conditions, promoting the development of venous diseases, such as varicosities [54]. Fragmentation of the elastic membranes and disturbed contact of elastic fibers with smooth muscle cells are important factors in the mechanical weakness and morphological deformation of varicose venous segments [55–58]. Accordingly, enhanced elastase activity was noted in varicose saphenous vein specimens from elderly subjects [59]. In older patients, a general reduction in elastin content is evident in the venous wall [59]. This reduction is correlated with a lack of fibrillin-1 in some areas and with a disorganized pattern of cells expressing tropoelastin and fibrillin-1. A decline in elastin content is causally linked to the deformed morphology of varicose veins. These dilated, elastin-poor segments are alternating with nondilated segments with normal elastin and collagen content. Reduced expressions of the elastin precursor tropoelastin and of lysyl oxidase, the enzyme responsible for the cross-linking of mature elastic fibers was also demonstrated in older people [60]. One theory largely attributes the vascular aging process to the uncoupling of the smooth muscle elastin receptor which results in elevated elastase release [61]. These connective tissue rearrangements are accompanied by increased rigidity and reduced contractility of the wall with substantial hemodynamic consequences [18]. Expression of laminin and adhesion molecules increases in aging and likely contributes to the genesis of varicosities [43]. Diseased veins have marked alterations in the expression of extracellular matrix proteins and regulatory factors: while collagen I chain alpha 1 and alpha 2 and laminin beta-1, beta-2, and gamma-1 are upregulated, small leucine rich proteoglycans that control collagen fiber assembly are reduced in varicose veins [54].

### Age-related changes in biomechanics and hemodynamics in veins

The age-related cellular and molecular alterations described above induce substantial alterations in the geometry and biomechanics of the vein wall, and consequent functional changes [62]. The venous wall is rigid at higher physiological pressures, while it is distensible at lower pressures. Venous wall thickening and increased collagen-to-elastin ratio reduces lower limb venous distensibility in the supine position by 78% in elderly population [63]. Furthermore, aging reduces calf venous compliance by up to 40–45% as the efficiency of the calf muscle pump decreases in resting venous capacity increases in older adults [63–65]. Distensibility of the upper limb venous system also decreases by 38% with aging [63]. In aging, the distensibility of inner jugular vein decreases by 68% in supine body position but increases by 106% in erect body position [63, 66]. The maximum capacity of the internal jugular vein increases with aging and is more pronounced on the right side and in males [63]. In contrast, distensibility of axillary veins does not change significantly with age, which can be explained by their intramuscular location [63]. Age-related changes in venous distensibility and compliance can be attenuated by regular physical exercise [67].

The mechanisms responsible for orthostatic tolerance in humans affect mostly the venous system, which represents one of the major evolutionary challenges for our species. Beyond adaptation to the erect body position, veins control adaptation to altered blood volume. Local myogenic and humoral mechanisms as well as systemic hormonal and nervous system influence venous biomechanics [68]. Long-term gravitational adaptation leads to altered venous wall geometry, contractility, and innervation density as well as altered venous network [16, 68]. There is evidence that regulation of the aforementioned venous functions is affected by aging [63]. In saphenous veins of older adults, a reduced activity of postjunctional alpha2 adrenoreceptors was observed, which may adversely affect venoconstrictor reflexes [69]. Functional deterioration of venous adaptations substantially contributes to the proneness of elderly people for orthostatic hypotension, collapse of the circulation in exsiccosis, hemorrhage, and heat exposition. Of note, decreased sympathetic reflexes may be partially compensated for by the decreased compliance [70–73].

### Age-related alterations in venous valves

The essential role of deep vein valves in the development of the varicosity disease has been recognized by Moore [74]. Venous valves are bicuspid and are positioned in a valve sinus, which is a local widening of the venous wall. The area between a valve leaflet and the vessel wall is called the valve pocket [75, 76]. The two cusps are thicker at their attachment to the venous wall (termed the limbus). Microscopically, the luminalis zone is the part of the cusp close to the lumen and facing the circulating blood stream and consists one layer of endothelial cells [77]. Beneath this layer, there is a moderately thick, wavy elastic lamella, the continuation of the internal elastic lamella of the intima. The parietalis zone is the part of the cusp facing the vein wall of the sinus, and is lined by one layer of endothelial cells. The crypts lined by endothelium face the sinus with their bases and usually are found in irregular intervals. The parietalis zone consists of loosely arranged collagen fibers and connective tissue cells that is gradually replaced by a thick dense collagen starting after the age of 30 [77]. Aging crypts of the parietalis zone tend to become shallower and the thickness of the elastic lamellae increases slightly. From the sixth decade, the elastica itself becomes thicker in the aged and the fatty tissue extends from the adventitia into the media of the vein wall. Distal to the valve, there is a proliferation of subintimal connective tissue and elastic fibers with aging (termed endophlebohypertrophy) [77]. Overall, the venous valve becomes thickened and less flexible with increasing age resulting in blood flow disturbance, thus enhancing blood stasis in the valve sinus and increasing time of reverse flow after valve closure [77, 78]. Venous hypertension is a key factor in valvular remodeling [79]. The age-related thickening of venous valves is a result of alterations in valve cusp structure including increased collagen deposition [80, 81]. These structural changes lead to functional changes including diminished elasticity. Due to age-related blood stasis, there is increased risk for thrombosis. In patients with chronic venous disease, about 25% of valvular incompetence can be explained as a result of previous deep vein thrombosis [82]. The thrombus itself can mechanically damage the valve resulting in reflux of venous blood. Thicker and less flexible damaged valves in older adults are associated with deep vein thrombosis [83].

Usually, the inferior vena cava is without a valve. In about 70% of limbs, there is one valve in the common femoral vein the saphenofemoral junction that protects the saphenous axis against increases in intra-abdominal venous pressure [76, 84]. The femoral vein exhibits approximately 3 valves, the popliteal veins 1 to 3. Many more valves are present in the deep venous system in the lower extremity: 8 to 19 valves are located in each of the posterior tibial veins and 8 to 11 valves in both the anterior tibial and peroneal veins [75, 85, 86]. The number of valves in perforating veins ranges between 1 and 5; however, avalvular perforating veins are mainly located in the foot, hand, and forearm [75]. Approximately 7 valves are located along the entire length of the great saphenous vein [87]. The number of valves in varicose saphenous veins is significantly lower compared to nonvaricose ones [88]. The number of valves in Africans is higher comparing to whites that may account for the high prevalence (10–18%) of varicose veins in whites and the low prevalence (1–2%) of the condition in Africans [85].

## Venous thrombosis

In older adults, thrombotic risk is significantly increased [89] resulting in 1% per year incidence of venous thrombosis [90]. Deep vein thrombosis of the lower extremity is the most common form of thrombosis. Additionally, venous thrombosis can also occur in the superficial veins of the leg and also in other veins, such as veins of the upper extremity, liver, cerebral sinus, and retinal and mesenteric veins. The Worcester Deep Vein Thrombosis Study demonstrated that the incidence of both deep vein thrombosis and pulmonary embolism increases exponentially with age [91]. The mechanisms contributing to these age-related changes are multifaceted.

In aging, the endothelial cells exhibit pro-thrombogenic phenotypic changes [21] and the morphology of the venous wall is altered, forming recesses with low flow where activated clot factors and platelets can accumulate. Platelets and endothelial cells in older adults overexpress P-selectin, an inflammatory adhesion protein, contributing to a procoagulant state [92]. Aging may also be associated with shortened prothrombin time; increased plasma levels of FVII, FVIII, and vWF; and increased platelet counts [81] Increases in fibrinogen, factors VIII and IX, and other coagulation proteins, without a proportional increase in anticoagulant factors, likely contribute to the increased thrombosis risk [90].

Currently, over 35% of individuals aged 65 and older are obese (over 55% of Black women) and if the current trend continues, nearly half of the elderly population in the USA will be obese by 2030 [93, 94]. Obesity in older adults may heighten thrombotic risks as the adipose tissue is an important source of factors regulating thrombus formation including inflammatory cytokines and plasminogen activator inhibitor-1 (PAI-1) [95].

Older adults frequently have a sedentary life style [96], which exacerbates the risk of venous thrombosis. The venous compliance in the calf decreases as the muscular tone of the calf decreases with age. As a result, the function of the aged venous valves often become impaired leading to higher thrombotic risk [97, 98]. The number of vascular risk factors and prevalence of chronic diseases are higher in the elderly population, which also contributes to higher thrombosis risk [98]. Additionally, abnormalities of the coagulation system, either genetic or acquired, exacerbate thrombotic risk. Acquired hypercoagulable states (e.g., associated with cancer) are more common in older adults. Inherited thrombophilia is caused by a variety of genetic abnormalities in anticoagulant factors such as antithrombin (AT), protein C (PC), and protein S (PS), or coagulation factors such as prothrombin and factor V. Genetic abnormalities in anticoagulant factors (such as deficiencies of antithrombin, protein C and protein S) are found in < 1% of the population and often present with unusual clinical episodes and localization of venous thrombosis. In a large multicenter cohort study of familial thrombophilia (European Prospective Cohort on Thrombophilia, EPCOT), the annual rate of venous thrombosis was 8 per 1000 without a clear age effect [99]. However, the incidence of venous thrombosis in patients aged 45 years and older is higher (1–2% per year) in retrospective family studies [100, 101]. Genetic abnormalities in procoagulant factors (e.g., Factor V Leiden, leading to APC-resistance and prothrombin G20210A leading to elevated levels of prothrombin) are common variants with an overall incidence of carriers of 2–5% among Caucasians and they are found in 6–20% of patients with deep vein thrombosis [102–106]. The LITE (Longitudinal Investigation of Thromboembolism Etiology) study investigated the absolute risk of thrombosis for carriers of FV Leiden of different ages [107]. In subjects older than 45 years of age, FV Leiden led to a 4.6-fold increased risk of venous thrombosis (vs. noncarriers) [108]. Elevated levels of procoagulant factors (i.e., prothrombin (FII), FVIII, FIX, and FXI) are associated with the risk of thrombosis [109–111]. Regarding environmental factors, surgery, major trauma, immobilization, pregnancy, postpartum period, long-distance travel, and cancer are the main risk factors for deep vein thrombosis [112–115]. Hypercoagulability in patients with malignancy was described first by Armand Trousseau in 1865 [116]. Cancer may induce venous stasis, endothelial injury, and an imbalance of pro- and anti-thrombotic factors leading to a hypercoaguable state [115]. Immobilization and prolonged travel increases the risk of thrombosis 2- to threefold in older adults [114]. The RIETE (Registro Informatizado Enfermedad TromboEmbolica) registry is a large prospective multinational ongoing registry, designed to collect data of venous thromboembolism presentation, management, and outcomes from multiple centers in 24 countries [117]. In the RIETE registry of patients aged over 80 with venous thrombosis, it was found that they had been immobilized for more than 4 days and had chronic obstructive lung disease and heart failure [97].

The most serious complication of venous thrombosis is pulmonary embolization and paradoxical embolism leading to ischemic stroke. Paradoxical embolism resulting in ischemic stroke can occur in the case of a patent foramen ovale, present in about 20% of the population. When the right atrial pressure transiently exceeds the left atrial pressure, even small venous emboli can transmit the canal of patent foramen ovale. Post-thrombotic syndrome develops in approximately 25–60% of patients with acute lower extremity deep venous thrombosis depending on severity, chronicity, anatomic level of involvement, and efficacy of anticoagulation [118]. The most prominent clinical signs of post-thrombotic syndrome are leg swelling, pain, and skin alterations, and even skin ulceration.

Residual thrombus damages venous valves and obstructs outflow, the main etiologic factors for ambulatory venous hypertension, which has been shown to be significantly associated with an increased risk of post-thrombotic syndrome. The main pathomechanism leading to post-thrombotic syndrome is aseptic inflammation triggered by thrombus formation, which results in venous wall fibrotic remodeling. Additionally, thrombus formation via direct mechanical venous valve damage exacerbates venous valve incompetence, contributing to the development of post-thrombotic syndrome. The relationship between venous valve incompetence and thrombosis is bi-directional, as incompetent venous valves promote venous stasis and thereby thrombus formation [119]. Remodeling of the vein wall in the post-thrombotic syndrome is more extensive in elderly people [120]. Timely removal of the thrombus may improve deep venous flow and hence decrease the incidence of post-thrombotic syndrome. Patients treated with new oral anticoagulants (including dabigatran, rivaroxaban, apixaban, and edoxaban) or percutaneous endovenous intervention for lower extremity deep venous thrombosis showed lower incidence of post-thrombotic syndrome and reduced recurrent deep vein thrombosis and venous obstruction [121] [122]. Endovascular methods have been developed as an aggressive treatment for lower-extremity deep vein thromboses that can remove acute venous thrombus and facilitate stent treatment of underlying venous stenoses [123]. These involve catheter-directed thrombolysis and percutaneous mechanical thrombectomy, balloon venoplasty, iliac vein stenting, and manual aspiration [124]. The lysis of endovascular thrombus results is a more rapid thrombus dissolution than systemic thrombolysis, thereby preserving valvular function [125]. The most important and most frequent complication of catheter-based interventions in patients with deep vein thrombosis is bleeding, mostly related to the use of thrombolytic agents [126].

## Chronic venous disease — varicose veins

 Chronic venous disease of the lower limbs is manifested as a progressive impairment of the venous circulation of the tissues [82]. The clinical signs of chronic venous disease range from edema, venous eczema, hyperpigmentation of skin, and lipodermatosclerosis (induration caused by fibrosis of the subcutaneous fat) to varicose veins and venous ulcers [82]. Chronic venous disease can be graded according to the descriptive Clinical, Etiological, Anatomical, Pathophysiological (CEAP) classification [82]. The clinical signs are categorized into seven classes (designated C0 to C6) according to severity [82]: no visible or palpable signs of venous disease (C0), telangiectasias or reticular veins (C1), varicose veins (C2), edema (C3), pigmentation or eczema (C4a), lipodermatosclerosis or atrophie blanche (C4b), healed venous ulcer (C5), and active venous ulcer (C6). Varicose veins are dilated, thickened, elongated, and twisted blood vessels, whose ability to control organ blood flow is impaired. Varicosities are present from class 2 of chronic venous disease. Severe chronic venous disease (C4 to C6) is termed “chronic venous insufficiency,” which is characterized by the presence of skin alterations in addition to varicose veins [82].

Advanced age, obesity, family history, and a sedentary lifestyle represent major risk factors for the development of chronic venous disease [82]. Severity of chronic venous disease progresses with advanced age [82]. The Framingham Study showed that the incidence of chronic venous disease is higher among women than men [4]. Women with varicose veins are more often obese, have lower levels of physical activity, and have higher systolic blood pressure. Men with varicose veins are characterized by lower levels of physical activity and higher smoking rates [4]. Although the pathomechanism of chronic venous disease is not entirely known, it is characterized with venous hypertension, venous reflux, and venous wall inflammation and fibrosis that can progress in a vicious circle of inflammation resulting in further progression of venous hypertension, venous reflux, and production of inflammatory mediators [82]. In the Edinburgh Vein Study, venous reflux increased the risk of developing varicose veins, especially when combined deep and superficial reflux was present [4, 8].

The mechanisms by which aging exacerbates progression of chronic venous disease are multifaceted. There is a general thickening of the venous wall with aging, even without the presence of reflux or venous hypertension [127]. Spatial heterogeneity in PDGF production within the aged venous wall has been causally linked to tortuosity [128]. Chronic venous hypertension initiates a range of pathophysiologic changes in the venous wall and surrounding tissues including inflammation, and increased permeability of endothelium. The resulting accumulation of fibrin and hemosiderin in the perivascular tissues acts to exacerbate inflammation and promote collagen synthesis by fibroblasts leading to venous wall thickening and remodeling. Tissue hypoxia leads to apoptosis and extracellular changes [129]. Aging-induced inflammation in veins is associated with elevated cytokine production [130] and increased MMP activation, which likely play a critical role in the pathogenesis of chronic venous disease [131, 132].

### The effects of sex hormones on venous diseases

Sex differences in the pathogenesis of venous diseases have been extensively studied [133]. Female sex is associated with greater risk factors for varicosis [133]. Lower limb venous pressure depends on the degree of calf muscle mass and activity and body mass index. Females have lower resting venous pressures because females, in general, have smaller calf size than males [134, 135]. The progression of chronic venous disease in the Edinburgh Vein population-based cohort study did not differ by sex, but family history of varicose veins or deep venous thrombosis increased the risk of disease progression [136]. The prevalence of deep vein thrombosis was higher in males. Similarly, the Austrian Study of Recurrent Venous Thromboembolism showed that men had a 3.6-fold higher risk of recurrent venous thrombosis than women [137, 138]. There are strong data suggesting that both estrogen and testosterone signaling pathways modulate biological processes involved in venous thrombosis [139–141]. Important in that regard is that women using exogenous estrogens either as contraceptives or as post-menopausal hormonal replacement have a higher risk of venous thrombosis [142–144].

## Role of veins in vascular cognitive impairment in aging

There is increasing evidence supporting an important role of age-related functional and structural alterations in cerebral veins in the pathogenesis of vascular cognitive impairment and dementia (VCID) [145]. Pathophysiological consequences of aging-induced dysregulation of the cerebral venous circulation potentially include disruption of the blood–brain barrier, development of cerebral microhemorrhages of venous origin, altered production of cerebrospinal fluid, glymphatic dysfunction, and dysregulation of cerebral blood flow [145].

Aging is known to alter the structure of cerebral veins, resulting in increased collagenosis [146, 147]. Increased venous collagenosis was demonstrated in brains with manifest leukoaraiosis [147], suggesting that pathological remodeling of the venous wall may contribute to the genesis of white matter lesions [148]. Recent studies started to determine how imaging alterations of deep medullary veins, small vessel disease, and cognitive impairment in older adults associate [149, 150]. There is emerging evidence that the increased diameter of the internal cerebral veins and of the basal veins of Rosenthal in older adults associate with regional white matter disease [150]. Periventricular venous collagenosis was reported to associate with white matter hyperintensities in both AD patients in older adults without AD pathologies [148]. In the aged, brain venules often exhibit increased tortuosity [151, 152]. It has been proposed that venular tortuosity may be an early neuroimaging marker of small vessel disease and may correlate with white matter hyperintensities and/or cerebral microhemorrhages [152]. A recent brain imaging study comparing deep medullary veins showed that patients with early Alzheimer’s disease also exhibit increased venular tortuosity [153]. The mechanisms contributing to exacerbated venous tortuosity in the brain are likely multifaceted and, based on analog mechanisms manifested in the peripheral venous circulation, may include elevated cerebral venular pressure (similar to the role of increased venous pressure in formation of varicose veins in the lower extremities [154]), altered elasticity of the vascular wall, degenerative changes of the media, and pathological remodeling of the extracellular matrix and basal membrane [151].

Age-related structural alterations of the bridging veins, which connect the superficial venous network to dural sinuses, have an important role in subdural bleedings associated with traumatic brain injury in older adults [155]. Because of brain atrophy and consequential expansion of the subdural space, elevated mechanical tension is imposed on the bridging veins in older individuals [155, 156]. The resulting increased mechanical burden combined with the aging-induced decrease in the elasticity of the venous wall predispose these bridging veins to a mechanically induced rupture in response to even minor brain trauma, resulting in increased incidence of bleedings into the subdural space in the elderly even with minor trauma [155, 156].

There is ample evidence documenting aging-induced degenerative changes and pathological remodeling in venous valves [81], which potentially contribute to venous valve insufficiency associated with advanced aging [77, 78]. On the basis of our understanding of the pathogenesis of chronic venous insufficiency in the peripheral venous circulation, it can be predicted that aging-induced alterations in cerebral venous valves also contribute to valvular incompetence [157], promoting venous reflux and cerebral venous hypertension. Elevated cerebral venous pressure has been proposed to contribute to pathological processes including microhemorrhages of venous origin, blood–brain barrier disruption, and perivascular inflammation, all of which promote age-related cognitive decline [145, 158, 159]. When venous hypertension develops in the superior sagittal sinus, it also impairs the absorption of the cerebrospinal fluid.

The internal jugular vein valve, which is the only venous valve situated in the venous circulation between the heart and the brain, is critical for the prevention of retrograde flow of venous blood. A missing or damaged internal jugular vein valve may promote jugular venous reflux [160]. There is strong anatomical evidence that the internal jugular vein valve is often incompetent in older adults. With an incompetent internal jugular vein valve, increases in intrathoracic pressure due to Valsalva maneuvers for example result in jugular venous reflux [161]. The incidence of jugular venous reflux significantly increases with advanced age [162–167], as a consequence of aging-induced degenerative changes in the venous valves. Jugular valve insufficiency and jugular venous reflux likely contribute to various brain pathologies [168, 169], including intra-cerebral hemorrhages of venous origin [170].

Cerebral white matter hyperintensities (WMHs) are a common radiological finding on MRI imaging of the aging brain showing damage in the white matter regions near the lateral ventricles (“leukoaraiosis”) [171–174]. WMHs can be diagnosed on T2-weighted fluid inversion recovery (FLAIR) sequences, without significant hypointensity on T1 images. The clinical significance of WMHs stems from their association with vascular cognitive impairment [175–177] as well as Alzheimer’s disease [178–181] in older adults. In addition to the well-documented contribution of pathological alterations in the arterial circulation (e.g., microvascular consequences of arterial hypertension), there is also increasing evidence supporting the role of aging-induced venous pathologies in the genesis of WMHs [182]. A number of age-related venous abnormalities were shown to associate with WMHs [146, 182], including jugular venous reflux and increased cerebral venous pressure [183].

Cerebral microhemorrhages (CMHs, also known as “cerebral microbleeds”), which result from rupture of small intracerebral blood vessels, are highly prevalent in older adults [159]. CMHs were reported to contribute to the pathogenesis of cognitive decline [159, 184–192]. In addition to the well-characterized arteriolar origin of CMHs, there is emerging evidence that CMHs can also originate from the rupturing of small veins, venules, and capillaries [159, 193–195]. Studies linking the development of CMHs to the performance of Valsalva maneuvers [196] support this concept. During the Valsalva maneuver, intrathoracic pressure can increase over 150 mmHg [197], which can be transmitted to the cerebral venous circulation if the internal jugular vein valves are incompetent [161, 198–201]. It has been proposed that when the pressure in the thin-walled cerebral venules exceeds a critical limit, multifocal venous CMHs may ensue in older adults.

Research into the pathogenesis of cerebral amyloid angiopathy has been primarily focused on Aβ deposition in the wall of arterial vessels. Yet, there is also increasing evidence from preclinical studies and clinical investigations that veins and venules are also affected by accumulation of Aβ likely through impaired perivascular clearance [202, 203]. It has been proposed that venular amyloidosis exacerbates microvascular pathologies associated with AD and may promote the development of amyloid plaques in the brain parenchyma as well [203].

## Aging veins as grafts

A special issue associated with vein aging relates to the surgical application of venous grafts for arterial bypasses. Saphenous veins have been used for coronary artery bypass grafting for more than 50 years. The advantages of this approach include ease of access, ease of operation, sufficiency of length for transplantation, and shortness of harvest time [204]. Vein grafts are living conduits which respond to hemodynamical and other local environmental stimuli. The transposition of vein segments from venous low pressure and low flow circumstances to arterial high pressure and high flow environment results in structural and functional remodeling of the venous wall. This remodeling can be either physiological or pathological, but the underlying regulatory mechanisms are not well understood.

The venous tunica media layer may become damaged during bypass grafting [205]. Vascular smooth muscle cells convert from contractile to synthetic phenotype as a consequence of damage. Altered shear, circumferential, longitudinal, compressive, and pulsatile stresses induce smooth muscle migration and proliferation into the intima, and deposition of collagen and proteoglycans into the intima and tunica media leading to thickening of these two layers [206]. The inward/outward luminal and wall remodeling of venous grafts leads to “arterialized veins” with altered structural and biomechanical features as compared to “normal” veins. Early studies showed that the lumen of venous bypass grafts may increase by 25 to 75% due to this adaptive remodeling [207]. The time course of venous graft remodeling is relatively rapid, the majority of the luminal and wall remodeling of the graft occurs in the first month after implantation [207]. The PREVENT III and IV randomized controlled trials showed that 30–40% of coronary and lower extremity vein grafts develop significant stenosis within the first year following implantation [205, 208].

There is evidence that grafting with veins from older adults is less successful than with younger veins [209–211]. Potential mechanisms contributing to pathological remodeling of older vein grafts include the dysregulated expression of Notch-4 [212], Ephr-B4 [213], smooth muscle myosin heavy chain, calponin, TIMP-2 and TIMP-3, cytokeratin-8, and MMPs [44].

## Shared mechanisms of vascular aging affecting the venous system

The role of shared cellular and molecular mechanisms of aging in age-related alterations of the venous system has not been studied in detail and can be inferred from studies on arterial aging [9, 10, 24]. Here we highlight some of these critical shared mechanisms of vascular aging, which may be targeted in future experimental and clinical studies for prevention of age-related venous alterations.

### Increased oxidative and nitrative stress

Strong evidence implicates increased oxidative stress in vascular aging processes, including the genesis of endothelial dysfunction and pathological vascular remodeling [22, 25, 30, 214–223]. Vascular oxidative stress results in impaired bioavailability of NO and increased generation of the highly reactive oxidant peroxynitrite (ONOO^−^; the reaction product of NO and superoxide). There is evidence that aging results in increased presence of nitrotyrosine (a marker of increased ONOO^−^ formation) in endothelial cells obtained from the antecubital veins [22], consistent with the view that increased oxidative and nitrative stress is a critical feature of venous aging. NO exerts potent anti-inflammatory, anti-thrombotic, and anti-leukocyte adhesion effects; thus, reduction in NO, in addition to the direct pro-inflammatory effects of increased levels of reactive oxygen species, likely contributes to age-related venous pathologies. Increased vascular oxidative stress has also been linked to activation of matrix metalloproteinases (MMPs) and consequential disruption of the structural integrity of aged vessels.

### Increased cellular senescence

Cellular senescence is emerging as an important mechanism of aging-induced vascular impairment [224, 225]. Oxidative stress–induced DNA damage is an important mechanism contributing to cellular senescence. Senescent endothelial cells express a senescence-associated secretory phenotype (SASP), characterized by increased secretion of inflammatory cytokines, immune modulators, growth factors, and proteases. There are studies supporting the concept that endothelial senescence is also a feature of venous aging [226]. Future studies are needed to define the role of senescence and SASP factors in the pathogenesis of venous alterations in aging.

### Increased inflammatory status

There is a strong connection between aging and chronic sterile inflammation in the cardiovascular system [10, 25, 27, 28, 217, 227–231]. The mechanisms contributing to age-related sterile vascular inflammation are likely multifaceted. Importantly, increased production of ROS activates pro-inflammatory signaling pathways, including NF-κB [222], which promote endothelial activation and up-regulate expression of various pro-inflammatory paracrine mediators. Aged venous endothelial cells also exhibit a pro-inflammatory phenotype, including an increased activation of NF-κB [22, 232]. Additionally, senescent cells also contribute to vascular inflammation via their SASP. Heightened inflammatory status likely contributes to pathological remodeling of aged veins [11, 38].

### Increased vascular apoptosis

Apoptosis is an evolutionarily conserved cell death program which was shown to contribute to a range of vascular aging phenotypes [10, 233]. In the aged arterial system, there is an increased presence of apoptotic endothelial cells, which has been linked to impaired bioavailability of pro-survival NO, increased levels of the pro-apoptotic inflammatory cytokine TNFα, and/or increased mitochondrial oxidative stress [29, 234–236]. Apoptosis also likely contributes to the pathogenesis of age-related venous diseases. Increased presence of apoptotic endothelial and smooth muscle cells was documented in varicose veins removed from older patients [40]. Recent observations confirmed the presence of apoptotic cells in the venous wall in chronic venous insufficiency [237].

### Role of sirtuins

Sirtuins (including SIRT1, SIRT3) are NAD^+^-dependent protein deacetylases, which regulate important cellular pathways involved in regulation of mitochondrial energy metabolism, cellular metabolic processes, chromatin function, and gene transcription [238–241]. There is strong evidence suggesting that sirtuin activation exerts anti-aging effects in the arterial system [242–245]. NAD^+^ is a rate-limiting co-substrate for sirtuins. Cellular NAD^+^ levels are decreased in advanced aging [246, 247], at least in part, as a consequence of increased NAD^+^ utilization by overactivated PARP-1 enzyme [248]. Recent studies suggest that in mouse models of aging, treatments that boost NAD^+^ biosynthesis (e.g., administration of nicotinamide mononucleotide, a key NAD^+^ precursor [246]) can activate sirtuins and reverse aging-induced endothelial dysfunction in the arterial system [23, 34]. Initial evidence suggests that interventions that activate SIRT1 or SIRT3 likely exert beneficial effect on the aged venous system as well [249, 250].

### Impaired cellular stress resilience

Impaired cellular stress resilience (the impaired ability of vascular cells to counteract the deleterious effects of various molecular stresses and return to homeostasis) has been identified as a universal hallmark of the aging process. In young organisms in the presence of increased production of ROS, adaptive homeostatic mechanisms are invoked, including the Nrf2 (nuclear factor erythroid 2-related factor 2)-driven antioxidant defense pathways [251–253]. Nrf2 is a redox-sensitive transcription factor, which orchestrates the antioxidant response [254]. In the young vasculature, this adaptive homeostatic mechanism up-regulates the expression of antioxidant enzymes and proteins that repair ROS-induced macromolecular damage, thereby protecting cells against oxidative injury triggered by dietary and lifestyle factors (e.g., smoking), diabetes mellitus, and inflammation. Importantly, Nrf2 activation has been shown to protect venous endothelial cells from oxidative stress–mediated apoptosis and injury [255–259]. Nrf2 activation was also demonstrated to confer potent anti-inflammatory [260] effects. Aging is associated with Nrf2 dysfunction in the vascular system, exacerbating oxidative stress and its sequela, including increased inflammation and cellular senescence [251, 252, 261]. Age-related loss of oxidative stress resilience is thought to promote development of vascular pathologies [262]. Recent studies provide preliminary evidence that mutations in the Nrf2 pathway may associate with deep vein thrombosis [263]. Further studies are warranted to determine how age-related Nrf2 dysfunction contributes to the genesis of venous aging phenotypes and to investigate the potential beneficial venous effects of pharmacological Nrf2 activators.

### mTOR signaling

Cellular mTOR signaling is an important regulator of metabolic processes including autophagy. Reduced activity of the mTOR pathway is well-documented to regulate aging processes [264]. There is growing evidence that experimental inhibition of mTOR activity (e.g., by rapamycin) interferes with the pathogenesis of a range of age-related diseases [265, 266] and exerts anti-aging endothelial protective effects [266–273]. Preliminary evidence also implicates mTOR in the pathogenesis of chronic venous insufficiency [274]. DEPTOR (domain-containing mTOR interacting protein) is involved in mTOR signaling pathway as an endogenous regulator. Recent studies demonstrate that overexpression of DEPTOR results in marked phenotypic changes in human saphenous vein endothelial cells [275].

## Interventions for healthy vein aging

Results from the Framingham Study suggest that increased physical activity and weight control may help prevent varicose veins among adults at high risk [90]. For prevention of deep vein thrombosis in older adults, lifestyle changes are warranted including increasing physical activity, ceasing to smoking, and reducing excess bodyweight. Conservative and interventional treatments can improve health-related quality of life and diminish or delay symptoms and progression of chronic venous insufficiency. Compression stockings are used to relieve symptoms; however, the compliance to wear them is not always adequate. Venoactive drugs may slow progression of chronic venous disease, but in advanced clinical stages aggressive medical intervention is needed. Thromboprophylaxis in high-risk states, such as immobilization and surgery, is mandatory. Furthermore, temporary anticoagulant therapy is needed in the case of venous thrombosis, while in the case of recurrent venous thrombosis or predisposing factor, lifelong anticoagulation is recommended. With the accessibility of molecular technologies, the time is not far when point-of-care testing will become available to identify thrombophilic genetic disorders, including mutations affecting the function of cells in venous wall, leading to early and proper prevention. Very long chain n-3 fatty acids in the diet lower thrombotic tendency, and flavonoids decrease platelet aggregation [276]. Because of the high global prevalence, screening and education programs for general practitioners have high importance [129, 277]. Moderate physical exercise seems to be one of the most effective means of prevention [4, 67, 96].

## Perspectives

There is growing evidence supporting the paradigm of the plasticity of vascular aging, suggesting that vascular aging phenotypes can be reversed by pharmacological or dietary interventions [23, 226, 235, 278–280]. It is predicted that in the upcoming decade, interventional strategies using combination treatments targeting multiple vascular aging processes can be developed to promote vascular rejuvenation. These will likely also be effective in improving venous health and preventing the pathogenesis of venous diseases in older adults. Public health research should also investigate the determinants of pathological venous aging (including the interaction of genetic, environmental, lifestyle, dietary, and socio-economic factors). Critical areas of venous aging research include mechanistic investigations targeting the contributions of venous pathologies to age-related cognitive impairment and neurodegeneration.
